# Uptake and willingness to use PrEP among Chinese gay, bisexual and other men who have sex with men with experience of sexualized drug use in the past year

**DOI:** 10.1186/s12879-020-05024-4

**Published:** 2020-04-22

**Authors:** Zixin Wang, Phoenix K. H. Mo, Mary Ip, Yuan Fang, Joseph T. F. Lau

**Affiliations:** 1 Centre for Health Behaviours Research, JC School of Public Health and Primary Care, Faculty of Medicine, The Chinese University of Hong Kong, Hong Kong SAR, China; 2 Shenzhen Research Institute, The Chinese University of Hong Kong, Shenzhen, China; 3 Department of Early Childhood Education, Faculty of Education and Human Development, The Education University of Hong Kong, Hong Kong SAR, China

**Keywords:** Pre-exposure prophylaxis, Willingness to use, Gay, bisexual and other men who have sex with men, Sexualized drug use, China

## Abstract

**Background:**

Sexualized drug use (SDU) refers to use of any psychoactive substance before/during sexual intercourse. Chemsex is a subset of SDU, which is defined as the use of some specific psychoactive substances (methamphetamine, mephedrone, γ-hydroxybutyrate, ketamine and cocaine) before/during sexual intercourse. SDU and chemsex are prevalent among gay, bisexual and other men who have sex with men (GBMSM). This study investigated uptake and willingness to use pre-exposure prophylaxis (PrEP) among a sample of GBMSM in Hong Kong with experience of SDU in the past year.

**Methods:**

A total of 600 GBMSM were recruited by convenient sampling through outreaching in gay venues, online recruitment and peer referral. Participants completed a cross-sectional anonymous telephone interview. This study was based on a subsample of 580 GBMSM self-reported as HIV negative/unknown sero-status.

**Results:**

Of the participants, 82 (14.1%) and 37 (6.4%) had experience of SDU and chemsex in the past year. The prevalence of PrEP uptake was 4.0% among all participants and 14.6% among those with experience of SDU in the past year. Among GBMSM with experience of SDU in the past year who were not on PrEP (*n* = 70), 67.1% were willing to use daily oral PrEP in the next 6 months. Multivariate logistic regression models showed that positive attitudes toward PrEP (AOR: 2.37, 95%CI: 1.47, 3.82), perceived support from significant others to use PrEP (AOR: 9.67, 95%CI: 2.95, 31.71), and perceived behavioral control of using PrEP (AOR: 19.68, 95%CI: 5.44, 71.26) were significantly associated with higher willingness to use PrEP.

**Conclusion:**

GBMSM with experience of recent SDU are potentially good candidates of PrEP implementation. This group of GBMSM reported high prevalence of uptake and willingness to use PrEP. Perceptions related to PrEP based on the Theory of Planned Behavior were significantly associated with willingness to use PrEP.

## Background

The HIV epidemic is severe among gay, bisexual and other men who have sex with men (GBMSM) in China, as the overall HIV prevalence and incidence in this group are both high and increasing (9.9% and 5.6 per 100 person-year in 2016) [1, 2]. In Hong Kong, China, where the study was conducted, the HIV prevalence and incidence among GBMSM was 6.54% and 1.0 per 100 person-year in 2017, respectively [3, 4]. Out of the 681 new HIV cases in 2017, 63% acquired it via homosexual or bisexual contact [3].

In line with numerous published studies, psychoactive substances were defined as the following: 1) ketamine, 2) methamphetamine, 3) cocaine, 4) cannabis, 5) ecstasy, 6) Dormicum/Halcion/Erimin 5/non-prescription hypnotic drugs, 7) heroin, 8) cough suppressant (not for curing cough), 9) amyl nitrite (popper), 10) GHB/GBL (γ-hydroxybutyrate), 11) 5-methoxy-N, N-diisopropyltryptamine (Foxy), and 12) mephedrone [5–7]. Sexualized drug use (SDU) refers to the use of any of the abovementioned psychoactive substances before/during sexual intercourse [8]. Chemsex is considered as a subset of SDU, which is commonly defined as the use of some specific psychoactive substances (methamphetamine, mephedrone, γ-hydroxybutyrate (GHB/GBL), ketamine and cocaine) before/during sexual intercourse [5–7]. These psychoactive substances were mainly used to facilitate, initiate, prolong, sustain and intensify sexual encounter [8, 9].

A high prevalence of SDU was reported among GBMSM in the United States (43%), Australia (54%), and Western Europe (18–54%) [5, 6, 10–13]. The rates were slightly lower in Asia, ranged from 28% in mainland China, 18% in Thailand, 14% in Vietnam, to 7% in Malaysia [14]. Worldwide, the prevalence of chemsex ranged from 3 to 35% among GBMSM [7, 13, 15]. A recent study reported that 12% of GBMSM in Hong Kong had SDU in the past 6 months (excluding the use of amyl nitrite alone) [16]. Psychoactive substances adversely affect users’ capacity to perceive and respond to risks during sexual encounters, leading to high-risk sexual practices (e.g., condomless anal intercourse (CAI), group sex, fisting, etc.) [17] and hence HIV and other sexually transmitted infections (STI) [7]. However, there was a lack of effective behavioral interventions in reducing psychoactive substance use and sexual risk behaviors among GBMSM with experience of SDU/chemsex [18–21].

Pre-exposure prophylaxis (PrEP) is a potentially risk reduction measure for GBMSM with experience of SDU/chemsex, which refers to the initiation of Tenofovir Disoproxil Fumarate/Emtricitabine before and during periods of HIV exposure among HIV-negative individuals in order to prevent HIV acquisition [22]. With good adherence, PrEP could significantly reduce the risk of HIV infection among various at-risk groups, including GBMSM [22] and drug users [23]. The World Health Organization (WHO) strongly recommends PrEP to all population at substantial risk of HIV infection [24]. Clinical trials did not identify any significant safety concerns with daily PrEP use among psychoactive substance users [23].

A systematic review suggested that prevalence of PrEP use among GBMSM varied from 2.5% in Australia, 2–5% in Western Europe, to 9–12% in the United States [25]. PrEP use is less common among GBMSM in Hong Kong (3.6% in 2017) [3]. Willingness to use PrEP among GBMSM also varied between 19.1 and 96.2%, with a pooled estimate of 57.8% [26]. Meta-analyses suggested that accessibility of PrEP and social stigma contributed to willingness to use PrEP among GBMSM, and country-specific factors were likely to affect willingness [26]. In Hong Kong, due to the high cost of obtaining formal prescription in private clinics (US$1000/month) and limited coverage of the PrEP demonstration project (70 participants), majority of local GBMSM PrEP users obtained the medication from overseas clinics or through online purchase [3]. Previous studies showed that about half of Hong Kong GBMSM in general were willing to use free PrEP [3, 27]. GBMSM with experience of SDU might have higher interest to use PrEP. One study in Hong Kong found that GBMSM with recent or ongoing SDU showed higher awareness of PrEP [28]. While another study in the United States reported that GBMSM using amyl nitrite had higher prevalence of PrEP use [29].

In order to develop effective intervention to promote PrEP, it is important to understand factors associated with willingness to use PrEP among GBMSM with experience of SDU. At least three quantitative studies investigated factors associated with willingness to use PrEP among high-risk drug users [30–32]. These studies had found a number of factors to be associated with willingness to use PrEP, including age, types and frequency of drug use, perceptions of HIV risk and PrEP (e.g., perceived effectiveness, would be less worried if on PrEP, concerns related to side effects, and confident to use/adhere to PrEP) [30–32]. However, these studies mainly targeted heterosexuals and injective drug users. The findings may not be generalized to GBMSM with experience of SDU. Only one qualitative [33] investigated reasons for PrEP use among substance-using GBMSM. The results suggested that being able to relax and not having to worry about getting HIV, and increased comfort and openness to have sexual relationship with HIV positive partners were facilitators of PrEP use among substance-using GBMSM. The participants of the qualitative study also expressed concerns that substance use/SDU would disrupt their daily routine and negatively affect their ability to take PrEP or adhere to daily PrEP [33]. These factors were considered by this study.

Theory-based interventions are more effective than non-theory-based ones [34]. In this study, we applied the Theory of Planned Behavior (TPB) [35] as the theoretical framework. The TPB postulates that behavioral intention to adopt a health-related behavior (e.g., use PrEP) is a strong predictor of actual behavior. In order to form such an intention, one would evaluate the pros and cons of the behavior (positive and negative attitudes), consider whether their significant others would support such behavior (perceived subjective norm), and appraise how much control one has over the behavior (perceived behavioral control). In recent published studies, the TPB has been successfully used to explain willingness to use PrEP among GBMSM in general [27], transgender women sex workers [36] and heterosexual population [37]. Moreover, many published studies also used the TPB to explain sexual behaviors [38–40], and utilization of HIV testing [41] and antiretroviral therapy [42].

In addition, mental health may be particular salient for PrEP use. Prevalence of mental health problems (e.g., depression, anxiety, and stress) are much higher among GBMSM than that of heterosexual men [43, 44]. Although acceptance of same-sex relationships is growing in many western countries, stigma toward homosexuals is still widespread in China [45]. As a result, previous studies reported high prevalence of mental health problems among GBMSM in China [46, 47]. Moreover, psychoactive substance use and SDU were associated with poorer mental health status among GBMSM [48–51]. Poor mental health status was found to be a barrier to start and adhere to PrEP [36, 52, 53]. For instance, anxiety symptoms were associated with lower willingness to use PrEP among transgender women sex workers in China [36]. Therefore, in this study, we investigated the associations between depressive/anxiety symptoms and willingness to use PrEP among GBMSM with experience of SDU in the past year.

To the best of our knowledge, there have been no study investigating willingness to use PrEP and associated factors among GBMSM with experience of SDU. Their prevalence of uptake and willingness to use PrEP as well as associated factors may be different from GBMSM without experience of SDU. To address these gaps, this study investigated PrEP uptake and willingness to use daily PrEP among GBMSM in Hong Kong with experience of SDU in the past year. Potentially associated factors were also investigated, including variables related to socio-demographics, service utilization, sexual behaviors, patterns of SDU, perceptions related to PrEP based on the TPB, and mental health status.

## Methods

### Participants and data collection

Data used in this study were derived from a cross-sectional study among GBMSM in Hong Kong from April to December, 2018. Participants were: 1) Hong Kong Chinese speaking men, 2) aged 18 years or above, and 3) had anal intercourse with at least one man in the last 12 months. Participants were recruited through multiple sources. Initially, a recent mapping exercise was conducted by the government and identified 12 gay bars and 16 gay saunas in Hong Kong. On approval of the owners, trained and experienced fieldworkers approached visitors in these venues at different time slots during weekdays and weekends. They briefed the prospective participants on details of the study and gave them an information sheet on site. Those who showed interest to join the study were asked some questions to screen their eligibility. Online outreaching to potential GBMSM was also used for study recruitment. The research team posted the information of this study periodically as new discussion topics on two popular gay websites in Hong Kong which are commonly used by GBMMS to seek sex partners. If prospective participants were interested in this study, they could contact the interviewers through private messaging or other means (e.g., WhatsApp, telephone, email, etc.). Recruitment was supplemented by peer referrals. Fieldworkers screened participants’ eligibility, guaranteed anonymity, right to quit at any time and that refusal would not affect chance to use any services. As approved by the ethics committee, verbal instead of written informed consent was obtained in order to maintain anonymity, and fieldworkers signed a form pledging that the participants had been fully informed about the study. Participants provided multiple contacts (mobile, emails, social media account, etc.) to fieldworkers and then made appointment to conduct a telephone interview.

During the interviews, trained interviewers confirmed participants’ eligibility over phone, and participants again provided consent to participate in the study. Interviewers then conducted telephone interview with the participants which took about 30 min to complete. At least five follow-up calls were made in different time slots during weekdays and weekends before considering the case as a non-contact. Incentive was provided to participants upon completion to compensate their time spent. A HK$50 supermarket or café coupon was mailed to an address provided by the participant, in an envelope containing no names, nor any information, about the study. Telephone numbers/addresses were cross-checked to avoid repetition. Ethics approval was obtained from the Survey and Behavioral Research Ethics Committee of the Chinese University of Hong Kong.

### Measures

#### Design of the questionnaire

A panel consisted of a public health researcher, an epidemiologist, one psychologist, one GBMSM and one non-governmental organization (NGO) worker formed to develop the questionnaire. Based on literature review and informal consultation involving all panel members, a tentative list of potential measurement was drafted. The panel members then rated the relevancy and importance on a pool of items in the list. A questionnaire was drafted and pilot among five GBMSM selected by purposive sampling. The five GBMSM commented on the relevancy, wording and content of the draft questionnaire. Based on comments made by GBMSM and all panel members, revision was made accordingly. The semi-final questionnaire was then pilot among another five GBMSM to seek further comments on wording and relevancy. All panel members then finalized the questionnaire. Participants of the pilot study did not take part in the actual survey.

#### Socio-demographics, utilization of HIV prevention services, sexual behaviors and mental health status

Participants were asked to report on socio-demographics, sexual orientation, HIV prevention services utilization, and history of STIs other than HIV. Queried sexual behaviors included anal intercourse with regular and non-regular male sex partners, CAI with men, and multiple male sex partnerships in the last year. Regular male sex partners (RP) were defined as lovers/stable boyfriends, while non-regular male sex partners (NRP) were defined as casual sex partners and/or male sex workers. Measurements of these variables had been used some published studies targeting GBMSM in Hong Kong [27, 54].

Probable depression was measured by validated Chinese version of the Center for Epidemiologic Studies Short Depression Scale (CES-D-10) [55], which has been widely used in studies targeting GBMSM [56, 57]. Scores ≥10 indicated presence of clinically significant depressive symptoms (range: 0–30) [55]. Anxiety symptoms were measured by validated Chinese version of the 7-item Generalized Anxiety Disorder Scale (GAD-7) [58]. A cut-off score of 15 is recommended to define severe anxiety [58]. In this study, the Cronbach alpha of the CES-D-10 and the GAD-7 was 0.893 and 0.930, respectively.

#### Patterns of SDU

Participants with experience of SDU in the past year were asked to report on the details about SDU, including: 1) types of psychoactive substance used during SDU in the past year, 2) poly-use of psychoactive substances during SDU in the past year, 3) time since the first episode of SDU, 4) frequency of SDU in the past year, 5) CAI during SDU in the past year, and 6) utilization of drug cessation/rehabilitation services in lifetime. Queried details of their most recent episode of SDU included number of people involved, use of alcohol and erectile dysfunction drug, and presence of group sex and CAI.

#### PrEP use and willingness to use PrEP

Participants who were currently on PrEP were asked about sources of PrEP, methods of PrEP use (i.e., daily PrEP, on-demand PrEP, and holiday PrEP: using PrEP either daily or on-demand only in some period of time). Daily PrEP users were asked about whether they had missed more than three doses of PrEP within a week in the past month. Such measurement of adherence was commonly used in published studies [59].

Participants with experience of SDU in the past year who were not on PrEP were briefed with the following: *“PrEP is a strategy that promotes taking oral antiretroviral drugs to prevent HIV infection among HIV-negative individuals. PrEP is strongly recommended by the WHO as an additional HIV prevention strategy for MSM. You are required to take PrEP once every day when you are using it in order to achieve its effect in preventing HIV infection. Daily use of oral PrEP could reduce risk of HIV infection by 92%. PrEP has possible side effects such as nausea, vomiting and headache”.* They were then asked whether they were willing to take a once-daily oral pill as PrEP in the next 6 months (Response categories: 1 = definitely not, 2 = probably not, 3 = neutral, 4 = probably will, 5 = definitely will). Responses were then dichotomized. Willingness to use daily PrEP was defined as “probably will” or “definitely will”. Such a definition has been commonly used in previous studies [27, 60, 61]. For those with willingness to use PrEP, they were further asked about the maximum amount (in HK$) they were willing to pay per month for using daily PrEP.

#### Perceptions related to PrEP based on the TPB

Two scales based on the TPB were constructed for this study, they are the 3-item Positive Attitude Scale and the 5-item Negative Positive Scale (response categories: 1 = disagree, 2 = neutral, 3 = agree). The Cronbach’s alpha for the Positive Attitude Scale and the Negative Attitude Scale was 0.648 and 0.747, single factors were identified by explanatory factor analysis, explaining for 56.6–61.6% of the total variances. We extracted one item from the validated Subjective Norm Scale (i.e., ‘people who are important to you will support you to use PrEP’; response categories: 1 = disagree, 2 = neutral, 3 = agree) [27] to measure perceived subjective norm related to PrEP. In addition, one item was extracted from the validated Perceived Behavioral Control Scale with some modification to measure perceived behavioral control related to PrEP use (i.e., ‘In general, you are confident in taking PrEP every day in the next six months’; response categories: 1 = disagree, 2 = neutral, 3 = agree) [27].

### Statistical analysis

Using SDU in past year (among all participants) and willingness to use PrEP (among participants with experience of SDU in the past year who were not on PrEP) as the dependent variables, crude odds ratios (ORu) of independent variables of interest were obtained by logistic regression models. Two summary multivariate logistic regression models were fit for these two dependent variables, using independent variables with *p* < 0.05 in univariate analysis as candidates. Adjusted odds ratios (AOR) and respective 95% confidence interval (CI) were obtained from the analyses. SPSS version 21.0 was used for data analysis, with *p* values< 0.05 taken as statistically significant.

## Results

### Socio-demographics, HIV prevention service utilization, sexual behaviors and mental health status

Out of 1131 prospective participants being approached through outreach in gay venues (*n* = 211), online recruitment (*n* = 607) and peer referral (*n* = 313), 906 showed interest to join the study and left their contact information. All these 906 participants were successfully contacted, 711 were screened to be eligible. Of eligible participants, 600 provided verbal informed consent and completed the telephone interview. The main reason for not providing informed consent was lack of time to complete the survey (*n* = 71), while the other 40 refusals did not specify their reason. This study was based on 580 GBMSM self-reported to be HIV negative/unknown HIV sero-status.

Most participants were aged 18–30 years (56.6%), currently single (84.0%), full-time employed (84.5%), with monthly personal income of at least HK$20,000/month (57.5%), and had attained at least a college education (84.5%). Over half had tested for HIV (71.6%) and had used other forms of HIV prevention services (55.3%) in the past year. In addition, 19.5% self-reported a history of STI other than HIV. In the last year, 85.3 and 60.9% had anal intercourse with regular and non-regular male sex partners, respectively. A total of 39.5 and 69.3% participants reported CAI with men and multiple male sex partnerships, respectively. The prevalence of probable depression and probable case of severe anxiety was 35.9 and 6%, respectively. (Table 1).

**Table 1:** Characteristics of GBMSM with and without experience of sexualized drug use (SDU) in the past year

	All (*n* = 580)	Without experience of SDU in the past year (*n* = 498)	With experience of SDU in the past year (*n* = 82)	With vs. without experience of SDU in the past year	
	%	%	%	OR (95%CI)	AOR (95%CI)
**Socio-demographic characteristics**					
Age group					
18–30	56.6	57.6	50.0	1.0	
31–40	31.6	30.5	37.8	1.43 (0.86, 2.37)	
> 40	11.9	11.8	12.2	1.19 (0.56, 2.50)	**–**
Highest educational level attained					
Senior high or below	15.5	14.3	23.2	1.0	1.0
College or above	84.5	85.7	76.8	0.55 (0.31, 0.98)*	0.36 (0.19, 0.69)**
Current marital status					
Currently single	84.0	84.9	78.0	1.0	
Married/cohabited with a man	15.7	14.7	19.8	1.63 (0.91, 2.91)†	
Married/cohabited with a woman	0.3	0.4	0.0	N.A.	–
Monthly personal income (HK$)					
< 10,000	13.4	12.7	18.3	1.0	
10,000-19,999	29.1	28.1	35.4	0.87 (0.44, 1.74)	
20,000-39,999	36.7	37.8	30.5	0.56 (0.28, 1.13)	
40,000 and above	19.7	20.5	14.6	0.49 (0.22, 1.12)†	
Refuse to disclose	1.0	1.0	1.2	0.84 (0.09, 7.73)	–
Current employment status					
Full-time	83.6	85.1	74.4	1.0	1.0
Part-time/unemployed/retired/ students	16.4	14.9	25.6	1.97 (1.13, 3.43)*	1.37 (0.74, 2.56)
Sexual orientation					
Homosexual	90.7	90.4	92.7	1.0	
Bisexual	8.4	8.6	7.3	0.83 (0.34, 2.01)	
Heterosexual	0.9	1.0	0.0	N.A.	–
Channels of recruitment					
Outreach in gay venues	14.1	13.3	19.5	1.0	
Online recruitment	57.6	58.6	51.2	0.59 (0.31, 1.12)	
Peer referral	28.3	28.1	29.3	0.71 (0.35, 1.42)	–
**Service utilization**					
HIV testing in the last 12 months					
No	28.4	29.9	19.5	1.0	
Yes	71.6	70.1	80.5	1.76 (0.99, 3.14)†	–
Other HIV prevention services in the last 12 months (e.g., condom distribution, peer education, pamphlet and lectures)					
No	44.7	46.8	31.7	1.0	1.0
Yes	55.3	53.2	68.3	1.89 (1.15, 3.11)*	1.77 (1.03, 3.05)*
**History of sexual transmitted infections**					
History of sexually transmitted infections other than HIV					
No	80.5	82.9	65.9	1.0	1.0
Yes	19.5	17.1	34.1	2.52 (1.51, 4.21)***	1.72 (0.97, 3.07)†
**Sexual behaviors in the last 12 months**					
Had had anal intercourse with regular male sex partners (RP)					
No	14.7	13.5	22.0	1.0	1.0
Yes	85.3	86.5	78.0	0.55 (0.31, 0.99)*	0.90 (0.46, 1.73)
Had had anal intercourse with non-regular male sex partners (NRP)					
No	39.1	44.0	9.8	1.0	1.0
Yes	60.9	56.0	90.2	7.26 (3.43, 15.38)***	7.75 (2.30, 26.09)**
Condomless anal intercourse (CAI) with men					
No	60.5	63.7	41.5	1.0	1.0
Yes	39.5	36.3	58.5	2.47 (1.54, 3.98)***	2.46 (1.44, 4.21)**
Multiple male sex partnerships					
No	30.7	34.1	9.8	1.0	1.0
Yes	69.3	65.9	90.2	4.79 (2.26, 10.18)***	0.84 (0.25, 2.80)
**Use of pre-exposure prophylaxis (PrEP)**					
Currently on PrEP					
No	96.0	97.8	85.4	1.0	1.0
Yes	4.0	2.2	14.6	7.59 (3.23, 17.86)***	4.09 (1.56, 10.73)**
**Mental health status**					
Depressive symptoms					
No clinical depressive symptoms (CES-D-10 score < 10)	64.1	65.7	54.9	1.0	
Presence of clinical depressive symptoms (CES-D-10 score ≥ 10)	35.9	34.3	45.1	1.57 (0.98, 2.52)†	**–**
Anxiety					
No/mild anxiety (GAD-7 score < 15)	94.0	94.4	91.5	1.0	
Severe anxiety (GAD-7 score ≥ 15)	6.0	5.6	8.5	1.57 (0.66, 3.72)	**–**

*OR* Crude odds ratios

AOR: multivariate odds ratios obtained by multivariate logistic regression using variables in Table 1 that were found to be statistically significant in the univariate analysis as candidates

*95%CI* 95% confidence interval

† 0.05 < *P* < 0.10, * *P* < 0.05, ** *P* < 0.01, *** *P* < 0.001

*N.A*. Not applicable; --: not considered in the model

### Prevalence of SDU in the past year and associated factors

Among participants, 82 (14.1%) and 37 (6.4%) reported SDU and chemsex in the past year, respectively.

During SDU in the past year, popper (80.5%), Methamphetamine (31.7%), and GHB/GBL (31.7%) were the most commonly used psychoactive substances; 30.5% reported poly-use of psychoactive substances, and 39.3% reported CAI. Only 1.9 and 7.5% had ever utilized drug cessation/rehabilitation provided by governmental and non-governmental organizations. In the most recent episode of SDU, 21.7% involved more than two people, 14.5 and 30.1% used alcohol and erectile dysfunction drugs, 19.3 and 50.6% had group sex and CAI. (Table 2).

**Table 2:** Patterns of SDU and perceptions related PrEP among GBMSM with experience of SDU in the past year

	% / Mean (SD)
**Patterns of SDU**	(*n* = 82)
Types of psychoactive substance used during SDU in past year	
Ketamine	1.2
Methamphetamine	31.7
Cocaine	1.2
Cannabis	8.5
Ecstasy	3.7
Dormicum / Halcion / Erimin 5 / Hypnotic drugs (non-prescription)	0.0
Heroin	0.0
Cough suppressant (not for curing cough)	1.2
Amyl nitrite	80.5
GHB/GBL	31.7
5-methoxy-N, N-diisopropyltryptamine (Foxy)	4.9
Mephedrone	0.0
Poly-use of psychoactive substances in lifetime	
No	69.5
Yes	30.5
Time since the first episode of SDU	
< 1 year	40.2
1–2 years	15.9
3–5 years	15.9
> 5 years	28.0
Frequency of SDU in the past year	
1 episode/month	37.8
1–2 episodes/month	29.3
≥ 3 episodes/month	32.9
Condomless anal intercourse during SDU in the past year	
No	48.8
Yes	51.2
Drug cessation/rehabilitation services provided by governmental organizations	
No	97.6
Yes	2.4
Drug cessation/rehabilitation services provided by non-governmental organizations	
No	90.2
Yes	9.8
Details of the most recent episode of SDU	
Number of participants	
2	78.0
≥ 3	22.0
Alcohol consumption	
No	85.4
Yes	14.6
Use of erectile dysfunction drugs	
No	69.5
Yes	30.5
Group sex	
No	80.5
Yes	19.5
Condomless anal intercourse	
No	48.8
Yes	51.2
**Perceptions related to PrEP (among participants with experience of SDU in the past year who were not on PrEP)**	(*n* = 70)
Willingness to take once-daily oral pill as PrEP in the next six months after being briefed some facts of PrEP	
No (definitely not/probably not/neutral)	32.9
Yes (probably will/definitely will)	67.1
Willingness to pay (HK$ per month) for using once-daily oral pill as PrEP (among those with willingness to take PrEP in the next six months, *n* = 56)	
≤ 300	24.2
301–500	14.3
501–1000	18.6
1000–2000	21.4
2001–4000	17.1
4001–6000	2.9
6001–8000	1.4
> 8000	0.0
Perceptions related to PrEP based on the TPB	
Positive attitudes toward PrEP (% agree)	
PrEP can reduce your chance of HIV infection during SDU	81.4
PrEP would reduce your worry of HIV infection when having condomless sex during SDU	64.3
PrEP provides you more choice for HIV prevention	84.3
*Positive Attitude Scale*^*a*^	8.1 (1.3)
Negative attitudes toward PrEP (% agree)	
Psychoactive substances that are used during SDU would reduce the protective effect of PrEP	17.1
Psychoactive substances that are used during SDU would aggravate the side-effects of PrEP	30.0
Psychoactive substances that are used during SDU would make you forget to take PrEP	28.6
Daily use of PrEP would cause severe financial burden for you	80.0
You will be stigmatized by medical professionals when you are using PrEP-related services	37.1
*Negative Attitude Scale*^*b*^	10.2 (2.2)
Perceived subjective norm related to PrEP	
People who are important to you will support you to use PrEP	
Disagree/neutral	32.9
Agree	67.1
Perceived behavioral control to use PrEP	
In general, you are confident in taking PrEP every day in the next six months	
Strongly disagree/disagree/neutral	35.7
Agree/strongly agree	64.3

Sexualized drug use is defined as use of the following psychoactive substances before/during anal intercourse, including ketamine, methamphetamine, cocaine, cannabis, ecstasy, Dormicum/Halcion/Erimin 5/non-prescription hypnotic drugs, heroin, cough suppressant (not for curing cough), amyl nitrite (popper), GHB/GBL (γ-hydroxybutyrate), 5-methoxy-N, N-diisopropyltryptamine (Foxy), and mephedrone

^a^Positive Attitude Scale, three items, Cronbach’s alpha: 0.648, one factor was identified by explanatory factor analysis, explaining for 61.6% of the total variance. Higher score of the scale indicated more positive attitudes toward PrEP

^*b*^Negative Attitude Scale, five items, Cronbach’s alpha: 0.747, one factor was identified by explanatory factor analysis, explaining for 56.6% of the total variance. Higher score of the scale indicated more negative attitudes toward PrEP

In multivariate logistic regression model, participants who had utilized other forms of HIV prevention services (AOR: 1.77, 95%CI: 1.03, 3.05), had anal intercourse with NRP (AOR: 7.75, 95%CI: 2.30, 26.09), and CAI with men (AOR: 2.46, 1.44, 4.21) reported higher prevalence of SDU in the past year. As compared to participants without experience of SDU in the past year, those with such experience were more likely to use PrEP (AOR: 4.09, 95%CI: 1.56, 10.73). Higher education level was associated with lower prevalence of SDU in the past year (AOR: 0.36, 95%CI: 0.19, 0.69). (Table 1).

### PrEP uptake and willingness to use daily oral PrEP

Twenty-three participants (4.0%) were on PrEP at the date of the survey. Sources of their PrEP included online purchase (7/23, 30.4%), clinics in Thailand (6/23, 26.1%), clinics in Hong Kong (3/23, 13.0%), the ongoing PrEP demonstration project in Hong Kong (6/23, 26.1%), and friends (1/23, 4.4%). Majority of them (14/23, 60.9%) were taking daily PrEP, six (26.1%) were using on-demand PrEP, and three (13.0%) were on holiday PrEP. Five out of 14 daily PrEP users (35.7%) reported sub-optimal adherence to daily regimen in the past month.

Among 70 participants with experience of SDU in the past year who were not on PrEP, 67.1% were willing to use daily oral PrEP in the next 6 months. Among 28 participants who had chemsex in the past year who were not on PrEP, 19 (67.9%) showed willingness to use daily oral PrEP in the next 6 months.

### Factors associated with willingness to use daily oral PrEP in the next six months among GBMSM with SDU in the past year

Multivariate logistic regression model showed that three out of four constructs of the TPB were significantly and positively associated with willingness to use PrEP. They were: 1) the Positive Attitude Scale (AOR: 2.37, 95%CI: 1.47, 3.82), 2) perceived support from significant others to use PrEP (subjective norm) (AOR: 9.67, 95%CI: 2.95, 31.71), and 3) perceived behavioral control of taking PrEP every day in the next 6 months (AOR: 19.68, 95%CI: 5.44, 71.26). (Tables 3 & 4).

**Table 3:** Associations between background characteristics and willingness to take once-daily oral pill as PrEP in the next six months (among GBMSM with experience of SDU in the past year who were not on PrEP, *n* = 70)

	Row%	OR (95%CI)
**Socio-demographic characteristics**		
Age group		
18–30	53.2	1.0
31–40	57.8	0.55 (0.19, 1.59)
> 40	77.8	1.40 (0.25, 7.93)
Highest educational level attained		
Senior high or below	83.3	1.0
College or above	61.5	0.32 (0.08, 1.25)†
Current marital status		
Currently single	69.0	1.0
Married/cohabited with a man	64.3	0.81 (0.29, 2.22)
Monthly personal income (HK$)		
< 10,000	75.0	1.0
10,000-19,999	74.1	0.95 (0.20, 4.55)
20,000-39,999	63.6	0.58 (0.12, 2.80)
40,000 and above	44.4	0.27 (0.04, 1.70)
Current employment status		
Full-time	67.9	1.0
Part-time/unemployed/retired/ students	64.7	0.87 (0.27, 2.73)
Sexual orientation		
Homosexual	66.2	1.0
Bisexual	80.0	2.05 (0.22, 19.43)
Channels of recruitment		
Outreach in gay venues	73.3	1.0
Online recruitment	68.4	0.79 (0.21, 2.99)
Peer referral	58.8	0.52 (0.12, 2.32)
**Service utilization**		
HIV testing in the last 12 months		
No	50.0	1.0
Yes	72.0	2.60 (1.01, 7.18)*
Other HIV prevention services in the last 12 months (e.g., condom distribution, peer education, pamphlet and lectures)		
No	73.9	1.0
Yes	63.8	0.62 (0.21, 1.88)
Drug cessation/rehabilitation services provided by governmental organizations		
No	69.1	1.0
Yes	0.0	N.A.
Drug cessation/rehabilitation services provided by non-governmental organizations		
No	68.3	1.0
Yes	57.1	0.62 (0.13, 3.04)
**History of sexual transmitted infections/viral hepatitis**		
History of other sexually transmitted infections		
No	68.0	1.0
Yes	65.0	0.87 (0.29, 2.61)
**Sexual behaviors in the last 12 months**		
Had had anal intercourse with regular male sex partners (RP)		
No	68.8	1.0
Yes	66.7	0.91 (0.27, 3.02)
Had had anal intercourse with non-regular male sex partners (NRP)		
No	57.1	1.0
Yes	68.3	1.61 (0.33, 7.89)

OR: crude odds ratios

OR and 95% were bold for variables with *p* < 0.05

† 0.05 < *P* < 0.10, * *P* < 0.05

*N.A*. Not applicable

**Table 4:** Factors associated with willingness to take once-daily oral pill as PrEP in the next six months (among GBMSM with experience of SDU in the past year and were not on PrEP, n = 70)

	OR (95%CI)	AOR (95%CI)
**Patterns of SDU**		
Poly-use of psychoactive substances during SDU the past year		
No	1.0	
Yes	2.02 (0.58, 7.01)	–
Time since the first episode of SDU		
< 1 year	1.0	
1–2 years	1.00 (0.21, 4.77)	
3–5 years	1.14 (0.25, 5.33)	
> 5 years	0.59 (0.18, 1.96)	–
Frequency of SDU in the past year		
1 episode/month	1.0	
1–2 episodes/month	0.81 (0.25, 2.67)	
≥ 3 episodes/month	1.33 (0.39, 4.58)	**–**
Condomless anal intercourse in the past year		
No	1.0	
Yes	0.88 (0.32, 2.40)	**–**
**Perceptions related to PrEP**		
Positive Attitude Scale	2.30 (1.46, 3.62)***	2.37 (1.47, 3.82)***
Negative Attitude Scale	1.16 (0.91, 1.47)	–
People who are important to you will support you to use PrEP		
Disagree/neutral	1.0	1.0
Agree	9.14 (2.90, 28.77)***	9.67 (2.95, 31.71)***
In general, you are confident in taking PrEP every day in the next six months		
Strongly disagree/disagree/neutral	1.0	1.0
Agree/strongly agree	20.57 (5.75, 73.65)***	19.68 (5.44, 71.26)***
**Mental health status**		
Depressive symptoms		
No clinical depressive symptoms	1.0	
Presence of clinical depressive symptoms	0.88 (0.32, 2.40)	**–**
Anxiety		
No/mild/anxiety	1.0	
Severe anxiety	0.46 (0.08, 2.45)	**–**

OR: crude odds ratios

AOR: multivariate odds ratios obtained by multivariate logistic regression using variables in Tables 3 and 4 that were found to be statistically significant in the univariate analysis as candidates

*95%CI* 95% confidence interval

OR, AOR and 95% were bold for variables with p < 0.05

*** *P* < 0.001, −--: not considered in the model

## Discussion

Our findings showed that 4% of our sampled GBMSM were on PrEP, rate that was comparable to that of a representative GBMSM survey conducted in 2017 (3.6%) [3]. Our hypothesis that GBMSM with experience of recent SDU had higher interest to use PrEP was supported by the results, as they had much higher PrEP uptake than those without such experience. Mathematical models suggested that achieving 75% PrEP coverage among high-risk HIV-negative GBMSM in China would prevent 25.7% of new HIV infection among all GBMSM [62]. GBMSM with experience of recent SDU may be a priority group for future PrEP implementation. Strategies to increase PrEP access and coverage are hence needed for this group of GBMSM in Hong Kong.

Among GBMSM with experience of SDU in the past year who were not on PrEP, about 70% were willing to use daily PrEP in the next 6 months. The rate was higher than that of GBMSM in general in Hong Kong [27]. Data on willingness to pay suggested that the current market rate for obtaining PrEP in Hong Kong (HK$8000 per month) was not affordable for this group of GBMSM, as none of them were willing to pay such amount. Future PrEP implementation in Hong Kong should take potential users’ willingness to pay into consideration. Without affordable PrEP in Hong Kong, this group of GBMSM may seek cheaper PrEP from informal channels (e.g., online purchases or oversea clinics) [3]. Previous studies showed that informal GBMSM PrEP users often reported sub-optimal adherence, risk compensation, and not taking up required testing [63]. These issues threaten their safety.

To achieve high coverage of PrEP, health promotion is needed even after affordable PrEP is available in Hong Kong. Findings of this study provided some insights for promoting PrEP among Hong Kong GBMSM with experience of recent SDU. Those who had taken up HIV testing in the past year showed higher willingness to use PrEP. HIV testing and counseling may be an ideal setting to promote PrEP, as confirmed HIV-negative sero-status is a prerequisite for initiate PrEP, and users may already be motivated to take up HIV protective measures and should be more ready to use PrEP [64]. Local NGO may play an important role in future PrEP implementation, as they are main providers of HIV testing services for GBMSM in Hong Kong [65].

Our results also suggested that increasing positive attitudes toward PrEP, creating subjective norm supporting PrEP use, and enhancing perceived behavioral control of using PrEP are potential useful strategies to promote PrEP, as these factors were significantly associated with willingness to use PrEP. Health communication messages should emphasize PrEP is an effective strategy in preventing HIV during SDU. Future health promotion should also encourage GBMSM with experience of SDU to discuss PrEP with their significant others to obtain support. Simplification of the procedures to obtain PrEP and provision of gay-friendly services may be useful strategies to enhance perceived behavioral control of using PrEP. In contrast to our hypothesis, negative attitudes such as concerns about psychoactive substances would reduce effectiveness, increase severity of side effects or risk of non-adherence, or lead to stigmatization originated from service providers were not significantly associated with willingness to use PrEP. Removing these negative attitudes may not be useful strategies to promote PrEP in this group.

In addition, our study also showed that prevalence of SDU and chemsex among GBMSM in Hong Kong was lower than that of Western Europe, Australia, the United States and mainland China [5, 6, 10–14]. Similar to previous findings, SDU in the past year was associated with higher prevalence of anal intercourse with NRP and CAI with men. Among GBMSM with experience of SDU in the past year, many of them engaged in SDU frequently, and reported CAI and group sex during SDU. Since we did not assess adoption of biobehavioral or behavioral risk reduction strategies (e.g., negotiated safety, serosorting, etc.) during sexual encounters, CAI might not represent high risk of HIV/STI infection. Future studies investigating sexual behaviors during SDU should take adoption of these biobehavioral or behavioral risk reduction strategies into account.

This study was one of the first studies looking at PrEP use among Chinese GBMSM with experience of recent SDU. However, it had some limitations. First, the small sample size of GBMSM with experience of SDU in the past year was one major limitation of this study. Only 70 GBMSM with such experience were asked about willingness to use PrEP and perceptions related to PrEP. This was a pilot study providing some preliminary data among this group of GBMSM with particularly high risk of HIV infection. Second, we did not ask PrEP-related questions among participants without experience of recent SDU. We were not able to compare perceptions related PrEP between GBMSM with and without experience of recent SDU. Third, we did not ask whether participants used PrEP or adopted other behavioral strategies (e.g., negotiated safety) during sexual encounters. Without measuring adoption of biobehavioral or behavioral risk reduction strategies, CAI might not represent high risk of HIV/STI infection. Fourth, participants were recruited by non-probabilistic sampling in the absence of sampling frame. As compared to a representative GBMSM survey in Hong Kong [3], our participants reported lower level of CAI, but higher HIV testing rate. Fifth, we were not able to obtain characteristics of participants who refused to join the study; selection bias might exist. Moreover, the results were self-reported and were collected via telephone interview, social desirability bias might exist. The prevalence of SDU, chemsex, CAI with men and multiple male sex partnerships may be under-reported. Furthermore, we did not ask participants’ willingness to use on-demand PrEP [66]. The willingness to use PrEP may be underestimated. Finally yet importantly, this was a cross-sectional survey and could not establish causal relationship.

## Conclusion

GBMSM with experience of recent SDU are potentially good candidates of PrEP implementation. This group of GBMSM reported higher prevalence of PrEP uptake and willingness to use PrEP. Perceptions related to PrEP based on the TPB were significantly associated with willingness to use PrEP.

## Data Availability

All data generated or analyzed during this study are included in this published article.

## Abbreviation

GBMSM: Gay, bisexual and other men who have sex with men
HIV: Human Immunodeficiency virus
SDU: Sexualized drug use
CAI: Condomless anal intercourse
PrEP: Pre-exposure prophylaxis
NGO: Non-governmental organization
STI: Sexually transmitted infection
ORu: Univariate odds ratios
AOR: Adjusted odds ratios
CI: Confidence interval
SD: Standard deviation
